# P-1819. Hepatitis B Virus Reactivation After Resolved Infection: A 10-Year US Cohort Analysis

**DOI:** 10.1093/ofid/ofaf695.1988

**Published:** 2026-01-11

**Authors:** Alejandro De la Hoz, Erin M Conolly, Lindsey R Baden, Ann E Woolley, Ella Woehl

**Affiliations:** Mass General Brigham, Boston, MA; Brigham and Women's Hospital, Boston, Massachusetts; Brigham and Women's Hospital, Boston, Massachusetts; Brigham and Women's Hospital, Boston, Massachusetts; Brigham and Women's Hospital, Boston, Massachusetts

## Abstract

**Background:**

Hepatitis B virus (HBV) reactivation remains a clinically significant complication in patients with prior resolved infection, particularly in those undergoing immunosuppressive treatments. Although highly effective recombinant vaccines have improved HBV prevention, data on reactivation patterns and vaccine protection in the current era are limited. We aimed to identify the prevalence of HBV reactivation in our health system, patient characteristics of those with and without reactivation, and highlight the immune protection gaps to inform targeted prevention strategies.

**Table 1.**
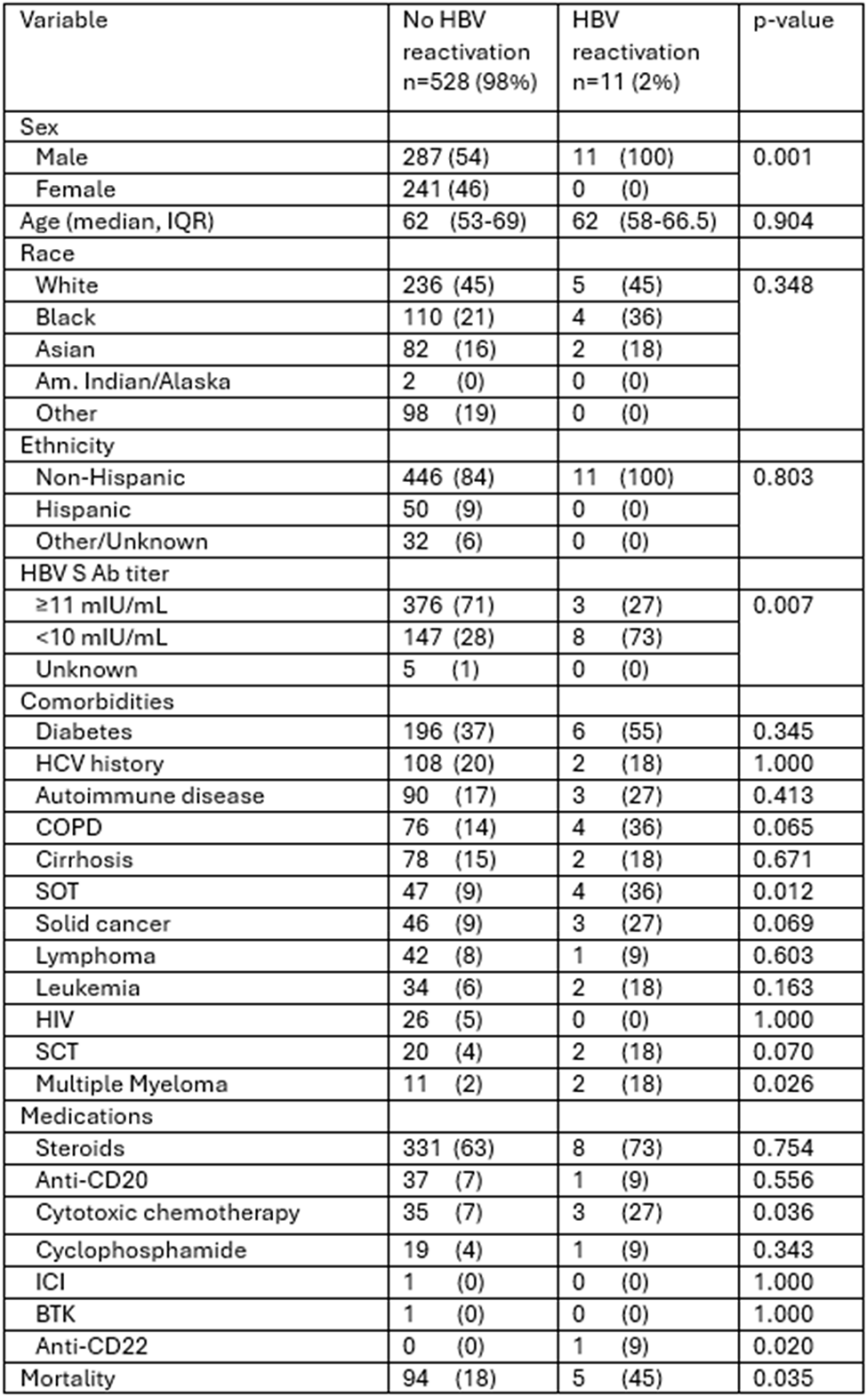
Characteristics of Patients with Past Hepatitis B Infection with and without Reactivation Am: American; IQR: interquartile range, n: number, HBV S Ab: hepatitis B S antibodies, COPD: chronic obstructive pulmonary disease, HCV: hepatitis C, HIV: human immunodeficiency virus, SOT: solid organ transplant, SCT: stem cell transplant, BTK: Bruton tyrosine kinase inhibitor, ICI: immune checkpoint inhibitor. Tests were 2-tailed, and a p-value < .05 was considered statistically significant.

**Methods:**

Retrospective cohort study from a tertiary hospital. We included patients with past HBV infection (positive core antibody and negative S antigen) who had subsequent viral load testing (HBV VL) between 01/2015 and 12/2024. We compared demographic and clinical characteristics of patients with and without HBV reactivation using descriptive statistics and univariate analyses.

**Results:**

Of 1,334 patients with past HBV infection with available data, 539 (40.4%) had subsequent HBV VL testing. The median age was 62 years (IQR 53-69). Most patients were male (n=298, 55%) and white (n=241, 45%). 11 (2%) patients had HBV reactivation. Non-protective antibody levels (< 10 mIU/mL) against the HBV S antigen were more frequent in patients with HBV reactivation (73% vs. 28%, p< 0.01). History of solid organ transplantation (36% vs. 9%, p< 0.05), multiple myeloma (18% vs. 2% p< 0.05), and cytotoxic chemotherapy use (27% vs. 7%, p< 0.05) were higher in the reactivation group (Table 1). All-cause mortality was higher in the reactivation group (45% vs. 18%, p< 0.05).

**Conclusion:**

HBV reactivation remains a clinical concern in the United States, particularly in immunocompromised hosts. The reactivation rate in our cohort aligns with prior reports. Most cases of HBV reactivation occurred in individuals with non-protective S antibody titers, underscoring the need for targeted immunization strategies in higher-risk groups. Recombinant vaccines offer a promising approach to reduce HBV reactivation risk in these vulnerable populations.

**Disclosures:**

Lindsey R. Baden, MD, Bill & Melinda Gates Foundation: Grant/Research Support|Bill & Melinda Gates Foundation: Collaboration of clinical trials conducted|COVID Vaccine Prevention Network: Collaboration of clinical trials conducted|Food and Drug Administration: Advisor/Consultant|HIV Vaccine Trials Network: Collaboration of clinical trials conducted|International AIDS Vaccine Initiative: Collaboration of clinical trials conducted|Johnson & Johnson: Collaboration of clinical trials conducted|Military HIV Research Program: Collaboration of clinical trials conducted|Moderna, Inc.: Collaboration of clinical trials conducted|National Institutes of Health: Advisor/Consultant|National Institutes of Health: Grant/Research Support|National Institutes of Health: Collaboration of clinical trials conducted|The Ragon Institute: Collaboration of clinical trials conducted|Wellcome Trust: Grant/Research Support

